# Adsorption of Serum Albumin onto Octacalcium Phosphate in Supersaturated Solutions Regarding Calcium Phosphate Phases

**DOI:** 10.3390/ma12142333

**Published:** 2019-07-23

**Authors:** Ryo Hamai, Kaori Tsuchiya, Osamu Suzuki

**Affiliations:** Division of Craniofacial Function Engineering, Tohoku University Graduate School of Dentistry, 4-1 Seiryo-machi, Aoba-ku, Sendai 980-8575, Japan

**Keywords:** octacalcium phosphate, bovine serum albumin, protein adsorption, degree of supersaturation

## Abstract

Octacalcium phosphate (OCP) has been shown to enhance new bone formation, coupled with its own biodegradation, through osteoblasts and osteoclast-like cell activities concomitant with de novo hydroxyapatite (HA) formation and serum protein accumulation on its surface. However, the nature of the chemical environment surrounding OCP and how it affects its metabolism and regulates protein accumulation is unknown. The present study examined how the degree of supersaturation (DS) affects the bovine serum albumin (BSA) adsorption onto OCP in 150 mM Tris-HCl buffer at 37 °C and pH 7.4, by changing the Ca^2+^ ion concentration. The amount of BSA adsorbed onto OCP increased as the DS increased. In addition, the amount of newly formed calcium phosphate, which could be OCP, was increased, not only by increases in DS, but also at lower equilibrium concentrations of BSA. The increased adsorption capacity of BSA was likely related to the formation of calcium phosphate on the adsorbed OCP. Together the results suggested that the formation of new calcium phosphate crystals is dependent on both the DS value and the adsorbate protein concentration, which may control serum protein accumulation on the OCP surface in vivo.

## 1. Introduction

Octacalcium phosphate (OCP, Ca_8_H_2_(PO_4_)_6_·5H_2_O) can act as a precursor to bone apatite crystals [1] based on its structural similarity to hydroxyapatite (HA) [2], solution chemistry [3,4] and theoretical solubility analyses [5,6]. When produced using specific synthesis conditions, synthetic OCP has a higher osteoconductivity compared to other calcium phosphate materials, such as non-sintered HA [7]. The osteoconductivity of OCP is related to its ability both to activate osteoblastic cells [8] and to recruit osteoclasts [9]. In vitro, direct or indirect cell contact with OCP materials enhances not only osteoblastic cell differentiation, but also osteoclast formation from their respective precursor cells [10,11,12]. Appositional bone formation after osteoclastic resorption in the physiological bone remodeling processes is known to involve apatite crystal nucleation and growth on bone matrix proteins when surrounding tissue fluid is supersaturated with respect to HA [13]. Thus, new bone formation onto OCP also likely includes such mineralization processes during crystal growth [14]. OCP exists in a metastable phase, and can undergo conversion to more thermodynamically stable HA under conditions of HA supersaturation [4]. Indeed, OCP can progressively convert to the apatitic phase when implanted into various bone tissues [7,15,16].

From the perspective of mineral crystal-matrix protein interactions, bone mineralization processes are typically accompanied by interactions between mineral crystals and proteins, including matrix proteins that are secreted by tissue-specific cells such as osteoblasts, in addition to circulating serum proteins [17] that together promote the formation of organized mineral/protein complexes in bone tissue [18]. Therefore, increased knowledge of how the surrounding tissue environment, in which materials are subjected to osteoclastic cellular phagocytosis and osteoblastic cellular mineralization, accompanying additional de novo calcium phosphate crystal deposition, regulates the mineral crystal-protein interaction.

Previous lectin histochemistry studies and proteome analyses showed that non-collagenous serum proteins accumulate around OCP crystals after implantation in mouse calvaria bone or upon immersion in rat serum in vitro [15,19]. New bone formation following OCP implantation onto mouse calvaria is likely initiated by constructs comprising complexes between OCP and non-collagenous proteins, as evidenced by ultrastructural observations of decalcified tissue specimens [7]. Ultrastructural observation of un-decalcified tissue specimens also confirms that de novo nanocrystals are deposited onto individual OCP crystals after their implantation onto mouse calvaria [20]. These results suggest that the environments where new bone forms on an OCP scaffold, or where OCP is being dissolved, could have certain saturation levels that can initiate new calcium phosphate crystal formation, regardless of the presence of serum proteins. However, there is limited information about whether saturation levels, which are determined by the concentration of ions, mainly calcium and inorganic phosphate, strengthen or weaken serum protein-OCP interactions, which can in turn affect the osteoconductivity of OCP materials.

Serum has been reported to be supersaturated with respect to HA and nearly saturated with OCP [21]. This finding is consistent with a previous observation that OCP that was implanted into rat calvaria bone defects tended to convert to the apatitic phase [10]. In addition to the possible ability of the osteoclast-like cellular resorption mentioned above to control saturation levels around OCP, the presence of mesenchymal stem cell-derived cells (ATDC5) with OCP increased calcium ion concentrations in the media, suggesting that OCP could be dissolved in response to some cellular activities [22]. OCP may encounter not only mesenchymal-derived cells, but also other cells such as inflammatory immune cells during bone formation after implantation [23,24] in the presence of serum proteins, thereby modulating the dissolution and re-precipitation of HA or possibly OCP onto the implanted OCP surface, which together yield the unique characteristics of OCP crystals at least in terms of protein adsorption.

The adsorption of bovine serum albumin (BSA) onto OCP can be described using a Langmuir-type monolayer adsorption model in which the adsorbate solutions are saturated with respect to the OCP phase [25]. Similar models were used to describe adsorption of saliva proteins and amino acids onto HA [26,27]. However, the appearance of calcium phosphate phases is thought to be governed by the degree of supersaturation (DS) [5], and the presence of other components, including proteins, may retard phase conversion and crystal growth [28,29,30].

Recently, OCP in granule form [31] and in composite with natural polymers [32] was clinically examined. Combining OCP with natural polymers [33,34] or synthetic polymers [35,36], and OCP coating onto metals [37,38], were also studied for possible use in clinical applications. OCP osteoconductivity reported within in vitro cellular experiments [10,11,12] and in vivo evaluations [7,9,10] has been confirmed for human oral bone tissues [31,32]. In the in vivo findings, bone formation mediated by OCP that initiates together with serum protein accumulation [7,15] may be involved in the clinically-observable bone regeneration, but little information is available to describe how serum proteins interact with OCP under physiological calcifying environments.

The present study seeks to investigate how the DS of the adsorbate solution regulates BSA adsorption onto OCP. In particular, this study focused on how the mechanistic relationship between calcium phosphate crystal phases and the adsorption capacity varies according to the DS in the environment around OCP implanted in vivo.

## 2. Materials and Methods

### 2.1. Preparation of OCP

Octacalcium phosphate (OCP) was synthesized by mixing calcium acetate and sodium hydrogen phosphate solutions following the wet synthesis method previously reported by Suzuki et al. [7]. Precipitates were separated from the solution and then washed with deionized water. After drying at 105 °C, the crushed precipitate was passed through a testing sieve (270 mesh) to obtain OCP granules < 53 µm in diameter and with a specific surface area of 16 m^2^·g^−1^ [25].

### 2.2. Adsorption Experiment

Tris-HCl buffer solution (150 mM) containing 1.5 mM Ca^2+^ and 1.0 mM inorganic phosphate (Pi) ion or 3.0 mM Ca^2+^ and 1.0 mM Pi ion (Ca1.5P1.0 and Ca3.0P1.0, respectively) was prepared by adding CaCl_2_·2H_2_O, K_2_HPO_4_, tris(hydroxymethyl)aminomethane (FUJIFILM Wako Pure Chemical Co., Osaka, Japan) and 1 M HCl solution into ultra-pure water to obtain solutions having different degrees of supersaturation (DS) with respect to HA and OCP. The buffer pH was adjusted to pH 7.4 using 1 M HCl at 37 °C (human body temperature). Subsequently, various amounts of bovine serum albumin (BSA, molecular weight: 66 kDa, Sigma-Aldrich, St. Louis, MO, USA) ranging from 0 to 1.5 mg·mL^−1^ were dissolved in each buffer.

To analyze the bovine serum albumin (BSA) adsorption behavior onto OCP under supersaturated conditions, 15 mg OCP granules were incubated in 3 mL of the abovementioned solutions containing BSA, with rotation mixing for 1 h at 37 °C. The mixture was centrifuged for 3 min at 3900 rpm, and the BSA concentration in the supernatant was measured using the Bradford method with a Coomassie Brilliant Blue (CBB) protein assay solution (Nacalai Tesque Co., Kyoto, Japan). The specimens were denoted as Cax Py BSAz, where x, y, and z refer to the concentration of Ca^2+^ (Ca), Pi ion (P), and BSA, respectively, in the solution used for the adsorption experiments.

### 2.3. Measurement of Ca^2+^ and Pi Ion Concentrations and Calculation of DS

Calcium and Pi ion concentrations in Ca1.5P1.0 or Ca3.0P1.0 solution containing BSA before (original solution) and after incubation with and without OCP for 1 h were measured using Calcium E and Phosphor C tests (Wako Pure Chemical Industries, Osaka, Japan), respectively. The free Ca ion concentration in the original solutions was also measured using a Ca ion-selective electrode (Orion 9720BNWP, Thermo Scientific, MA, USA) with a calibration curve produced using 150 mM KCl as the background electrolyte.

The DS values with respect to hydroxyapatite (HA), OCP and calcium hydrogen phosphate dihydrate (DCPD) in the solution were calculated to estimate the crystal growth in calcium phosphate phases using analytical values for Ca^2+^ and Pi ion in the supernatants. The DS value was typically calculated using analytical results for Ca^2+^ and Pi ion concentrations at 37 °C, pH 7.4 by taking the three mass balance values that account for Ca^2+^, Mg^2+^ and Pi ions, according to previous reports [39,40]. In this calculation, the presence of ion pairs (CaH_2_PO_4_^+^, CaHPO_4_^0^, MgHPO_4_^0^, CaHCO_3_^+^ and MgHCO_3_^+^) was considered, assuming that HCO_3_^−^ was present in the solution. However, the presence of BSA was not included, and the concentration of Mg^2+^ was set as approximately zero in the calculation. In addition, a background electrolyte of 150 mM Na^+^ as a substitute for 150 mM Tris was used in the calculation. The DS values were identified in terms of ionic activity products (IP) and solubility constants (*K*_sp_) with respect to HA, OCP and DCPD by Equation (1):

DS = (*IP*/*K*_sp_)^1/v^, (1)

where v is the number of ions in calcium phosphate (v = 9, 8 and 2, for HA, OCP and DCPD, respectively.) The solubility products for HA, OCP and DCPD were 7.36 × 10^−60^ (mol·L^−1^)^9^ [41], 2.51 × 10^−49^ (mol·L^−1^)^8^ [42] and 2.77 × 10^−7^ (mol·L^−1^)^2^ [43], respectively. DS values = 1, < 1, and > 1.0 indicated saturation, undersaturation, and supersaturation, respectively.

### 2.4. Characterization of OCP before and after Soaking in Supersaturated Solutions

OCP granules formed after incubation in Ca1.5P1.0 or Ca3.0P1.0 solutions alone, or those containing BSA, were repeatedly washed with ultra-pure water before lyophilization.

OCP granules before (original OCP) and after soaking in solutions containing BSA were characterized using a powder X-ray diffractometer (MiniFlex 600; Rigaku Electrical Co., Ltd., Tokyo, Japan). In the XRD analysis, the incident X-ray (monochromatic CuKα radiation, 40 kV and 15 mA) was used to scan the specimens at a rate of 1.0°·min^−1^ and step 0.02° intervals from 2θ = 3 to 60°.

The OCP granules before and after incubation were also analyzed with a Raman spectrometer (Model ApaRAMAN, LICIR Inc., Tsukuba, Japan) equipped with an optical microscope. The ratio areas for typical peaks corresponding to v_1_ PO4 of OCP at 596 cm^−1^ in the spectra were measured to compare the adsorption capacity of BSA onto OCP among the different DS values. In this measurement, the total peak area at 1600 cm^−1^ and 1652 cm^−1^ assigned to amino acid and amide I, respectively, was used as the BSA peak.

A curve fitting analysis of the Raman spectrum was also performed to estimate changes in the HPO_4_^2−^ content of OCP in the supersaturated solution without BSA. Peaks corresponding to ν PO_4_, ν HPO_4_ and P–O bend were separated in spectra of the specimens over the range 370–650 cm^−1^ using curve fitting software (Spectra Manager Version 2, Curve fitting Version 2.06.00, JASCO Co., Tokyo, Japan). The integrated intensity ratio of ν HPO_4_ to ν PO_4_ was then calculated as an indicator of the HPO_4_^2−^ content in OCP crystals in the supersaturated solution. In this analysis, curve fitting of Raman spectra for purified HA (APACERAM; HOYA Technosurgical Co., Tokyo, Japan) that was used as a reference specimen was performed using the same method as for OCP.

Bright field image and selected area electron diffraction (SAED) of OCP crystals before and after the incubation were obtained using a transmission electron microscope (FE-TEM; JEM-2100F, JEOL Ltd. Tokyo, Japan) equipped with an AMT CCD camera (AMT, Danvers, MA, USA) at 100 kV acceleration voltage.

### 2.5. Immersion Experiment for OCP Granules Pre-adsorbed to BSA in Supersaturated Solution

OCP granules soaked in Ca3.0P1.0 solution containing 0.25 or 1.5 mg·mL^−1^ BSA were incubated again in fresh Ca3.0P1.0 solution lacking BSA for 1 h. The Ca^2+^ and Pi ion concentrations in the Ca3.0P1.0 solution were then measured using the Calcium E and Phosphor C tests.

### 2.6. Statistical Analysis

Results are expressed as mean ± standard deviation (SD). One-way analysis of variance (ANOVA) was carried out to compare the means for more than three groups. If the ANOVA was significant, then a Tukey-Kramer multiple comparison analysis was performed. Student’s t test was also performed to compare means between two groups. The *p* values < 0.05 were considered statistically significant.

## 3. Results

### 3.1. Measurement of BSA Adsorption Capacity onto OCP in Solutions with Different DS

**Figure 1:**
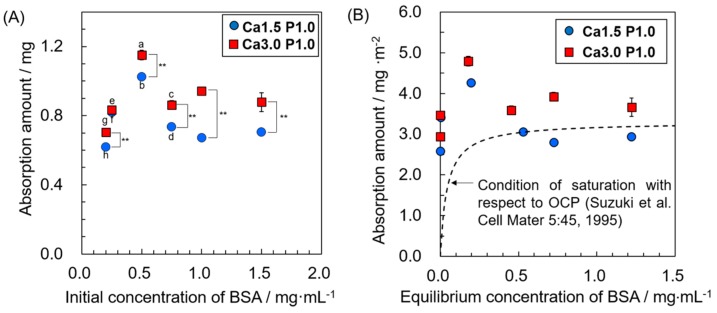
Adsorption isotherms of BSA onto octacalcium phosphate (OCP) plotted as adsorption amount as a function of initial BSA concentration (**A**) and adsorption amount per unit area of OCP as a function of BSA equilibrium concentration (**B**) in solutions containing 1.0 mM Pi ion and 1.5 or 3.0 mM Ca^2+^ (Ca1.5P1.0 and Ca3.0P1.0, respectively). Mean values were obtained from three independent experiments (n = 3). Significant difference (*p* < 0.01) for 0.20, 0.25, 0.75, 1.0 and 1.5 mg·mL^−1^ BSA in ^a^Ca3.0P1.0 and ^b^Ca1.5P1.0 solution; ^c^Significant difference (*p* < 0.01) for 0.20, 0.25 and 1.0 mg·mL^−1^ BSA in Ca3.0P1.0; ^d^*p* < 0.01 significantly difference from 0.20 and 0.25 mg·mL^−1^ BSA in Ca1.5P1.0; ^e^Significant difference (*p* < 0.01) from 0.20 and 1.0 mg·mL^−1^ BSA in Ca3.0P1.0; ^f^*p* < 0.01 significant difference for 0.20, 1.0 and 1.5 mg·mL^−1^ BSA in Ca1.5P1.0. ^g^*p* < 0.01 significant difference from 0.10 and 1.5 mg·mL^−1^ BSA in Ca3.0P1.0; ^h^*p* < 0.01 significant difference for 1.5 mg·mL^−1^ BSA in Ca1.5P1.0; **p < 0.01 significant difference between Ca1.5P1.0 and Ca3.0P1.0 solution at each BSA concentration. Dashed line indicates the adsorption isotherm of BSA onto OCP under saturation conditions with respect to OCP at 37 °C and pH 7.4 reported by Suzuki et al. [25], and is plotted using adsorption constants obtained from previous reports [25] based on the Langmuir equation (Equation (2)).

Absorption isotherms of BSA on OCP in the solution having different DS showed that the amount of BSA adsorbed onto OCP significantly increased with BSA concentrations up to 0.50 mg·mL^−1^ after incubation in the Ca1.5P1.0 or Ca3.0P1.0 solutions (Figure 1A). The adsorption amount of BSA significantly decreased as the initial BSA concentration increased from 0.50 to 0.75 mg·mL^−1^, at which point the amount adsorbed stabilized. The adsorption amount of BSA onto OCP was higher for Ca3.0P1.0 relative to Ca1.5P1.0 regardless of the initial BSA concentration. The absorption isotherms were plotted as the adsorption amount per surface area versus the equilibrium concentration of BSA (Figure 1B). The absorption isotherms rapidly increased at lower BSA equilibrium concentrations, and had local maximal values for the adsorption capacity in both Ca1.5P1.0 and Ca3.0P1.0.

### 3.2. Measurement of Ca^2+^ and Pi Ion Concentration in Buffer Solutions and Calculation of DS Values

Table 1 shows results for measurement of Ca^2+^ and Pi concentrations in the solution before (original solution) and after incubation without OCP granule soaking. After incubation for 1 h, the concentration of Ca^2+^ and Pi ions in both Ca1.5P1.0 and Ca3.0P1.0 containing BSA was within the range of that of the original concentration.

Changes in Ca^2+^ and Pi ion concentration in the solution were also measured during BSA adsorption onto OCP granules (Table 2). The ion concentrations in Ca1.5P1.0 and Ca3.0P1.0 decreased after the soaking of OCP granules. The calculated degree of supersaturation (DS) values in these solutions also decreased after incubation with OCP regardless of the initial DS and BSA concentration. The DS values were ordered as 10^8^–10^12^, 10^0^–10^1^ and 10^−1^ with respect to HA, OCP and DCPD, respectively, which indicated that, after incubation, these solutions were supersaturated and slightly supersaturated with respect to HA and OCP, respectively. The DS values with respect to HA and OCP tended to be higher for Ca3.0P1.0 compared to those for Ca1.5P1.0

**Figure 2:**
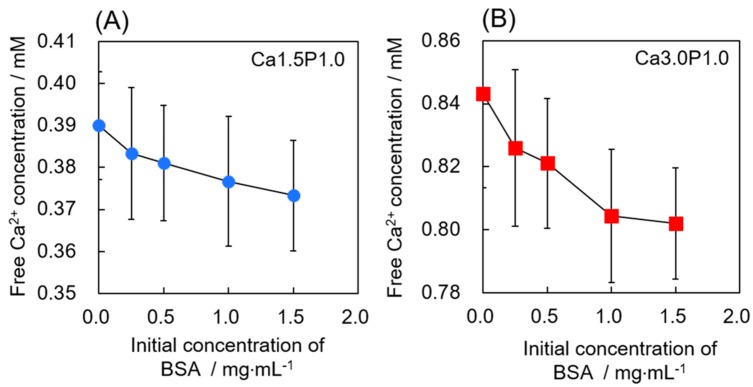
Change in free Ca^2+^ concentration from 0 to 1.5 mg·mL^−1^ BSA in Ca1.5P1.0 (**A**) or Ca3.0P1.0 (**B**) solutions. Mean values were obtained from three independent experiments (n = 3).

To evaluate the interaction between Ca^2+^ and BSA in the supersaturated solution, the free Ca^2+^ concentration in Ca1.5P1.0 and Ca3.0P1.0 in the absence of OCP granules was measured (Figure 2). The concentration of free Ca^2+^ tended to decrease with increasing BSA concentration for both Ca1.5P1.0 and Ca3.0P1.0, although the difference was not significant.

### 3.3. Characterization of OCP Granules before and after Soaking in Buffer Solutions Containing BSA

#### 3.3.1. XRD Analysis

**Figure 3:**
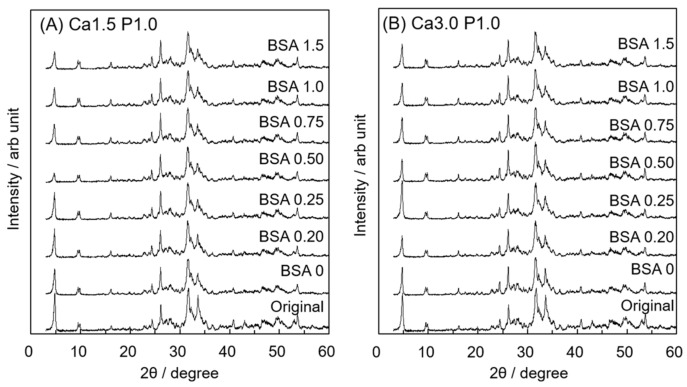
Powder XRD patterns of OCP before and after incubation with 0 to 1.5 mg·mL^−1^ BSA in Ca1.5P1.0 (**A**) or Ca3.0P1.0 (**B**) solutions.

Figure 3 shows XRD patterns of OCP before and after incubation in Ca1.5P1.0 or Ca3.01P1.0 solution containing BSA. The peaks corresponding to the (100), (010) and (002) planes of the OCP crystal structure were observed at 2θ = 4.7, 9.8 and 26° in the pattern of original specimens, respectively. Although the intensity of the peak corresponding to the (100) plane of OCP crystals slightly decreased, these characteristic peaks were also detected after incubation regardless of the DS value and BSA concentration of the solutions. On the other hand, no peaks attributed to other phases of calcium phosphate such as HA were observed in the diffraction patterns after the incubation.

#### 3.3.2. Raman Spectrometry

**Figure 4:**
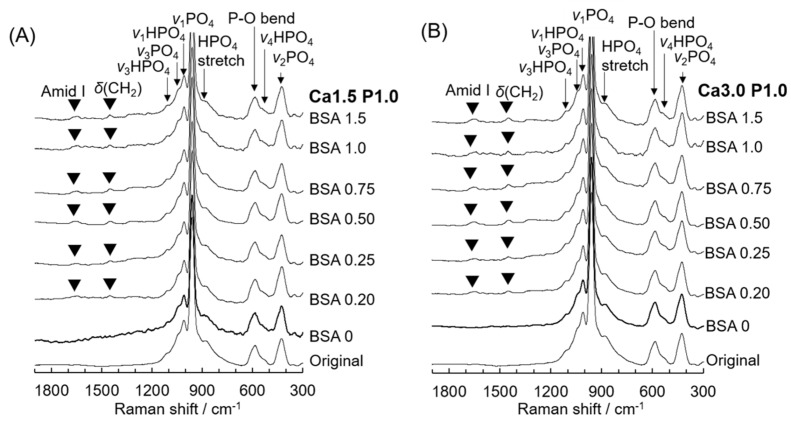
Raman spectra of OCP before and after incubation with 0 to 1.5 mg·mL^−1^ BSA in Ca1.5P1.0 (**A**) or Ca3.0P1.0 (**B**) solutions.

All Raman spectra of OCP granules before and after soaking in Ca1.5P1.0 and Ca3.0P1.0 containing various amounts of BSA showed peaks corresponding to ν_1_ PO_4_ and ν_1_ HPO_4_ for OCP at 957 and 1013 cm^−1^, respectively (Figure 4) [44]. Characteristic peaks for P–OH stretching and ν_3_ HPO4 stretching were also detected at 884 and 1116 cm^−1^, respectively, before and after incubation, and correspond to HP(5)O_4_^2−^ in the hydrated layer of the OCP crystal structure [44]. A peak attributed to δ(C–C) was observed after incubation in Ca1.5P1.0 and Ca3.0P1.0 containing BSA concentrations above 0.20 mg·mL^−1^.

**Figure 5:**
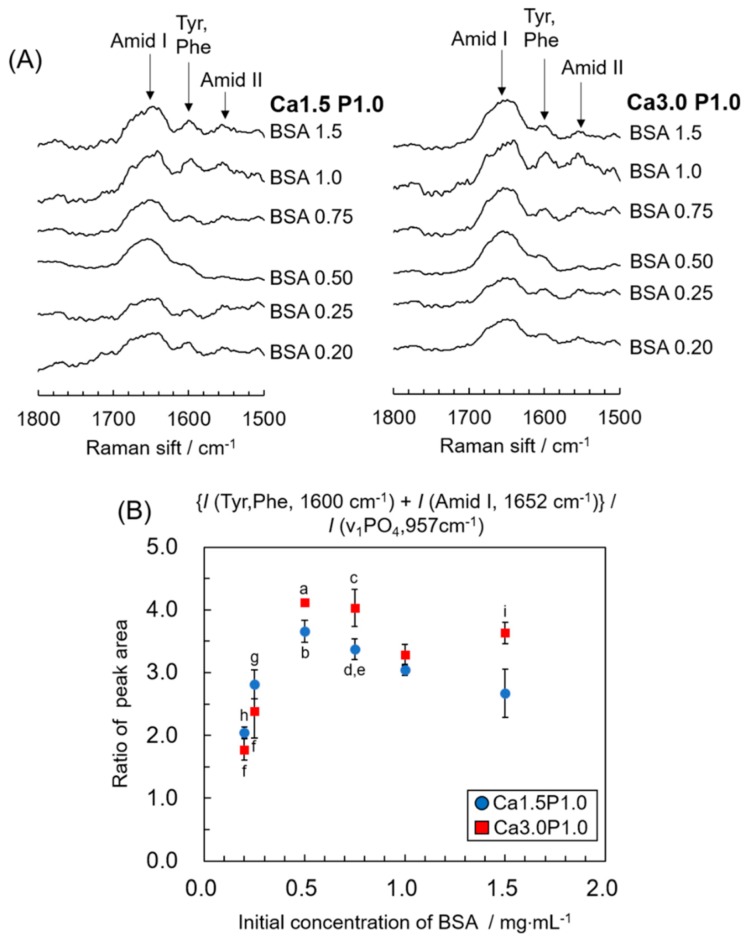
Magnified Raman spectra between 1500 to 1800 cm^−1^ (**A**) and change in integrated intensity ratio of the total of two BSA peaks at 1600 and 1653 cm^−1^ to the OCP peak at 957 cm^−1^ (**B**) after incubation with BSA solutions. ^a^*p* < 0.01 significant difference for 0.20, 0.25 and 1.0 mg·mL^−1^ BSA in Ca3.0P1.0; ^b^*p* < 0.01 significant difference for 0.20, 0.25 and 1.5 mg·mL^−1^ in Ca1.5P1.0 solution; ^c^*p* < 0.01 significant difference for 0.20, 0.25 and 1.0 mg·mL^−1^ BSA in Ca3.0P1.0. ^d^*p* < 0.01 significant difference between 0.20 and 0.75 mg·mL^−1^ BSA, and ^e^*p* < 0.05 significant difference from 1.5 mg·mL^−1^ BSA in Ca1.5P1.0; ^f^*p* < 0.01 significantly different for 1.0 and 1.5 mg·mL^−1^ BSA in Ca3.0P1.0; ^g^*p* < 0.05 significant difference for 0.20 mg·mL^−1^ BSA and ^h^*p* < 0.01 significant difference from 1.0 mg·mL^−1^ BSA in Ca1.5P1.0. ^i^*p* < 0.05 significant difference from 1.5 mg·mL^−1^ BSA in Ca1.5P1.0. Mean values were obtained from three independent experiments (n = 3).

In magnified Raman spectra between 1500 to 1800 cm^−1^ (Figure 5A), peaks attributed to amide III, the amino acids tyrosine and phenylalanine, and Amide I of BSA were observed at 1548, 1604 and 1653 cm^−1^, respectively, in the spectra for OCP granules after soaking in solutions containing between 0.25 and 1.5 mg·mL^−1^ BSA [45]. The change in the integrated intensity ratio of the total of the two peaks for BSA to the OCP peak at 957 cm^−1^ is shown in Figure 5B. The change in the ratio relative to the initial concentration of BSA was similar to the adsorption isotherm shown in Figure 1B. The ratio significantly increased at up to 0.50 mg·mL^−1^ BSA and then decreased at BSA concentrations between 0.50 mg·mL^−1^ and 1.5 mg·mL^−1^. In addition, the ratio tended to be higher for Ca3.0P1.0 compared to Ca1.5P1.0 at each BSA concentration.

**Figure 6:**
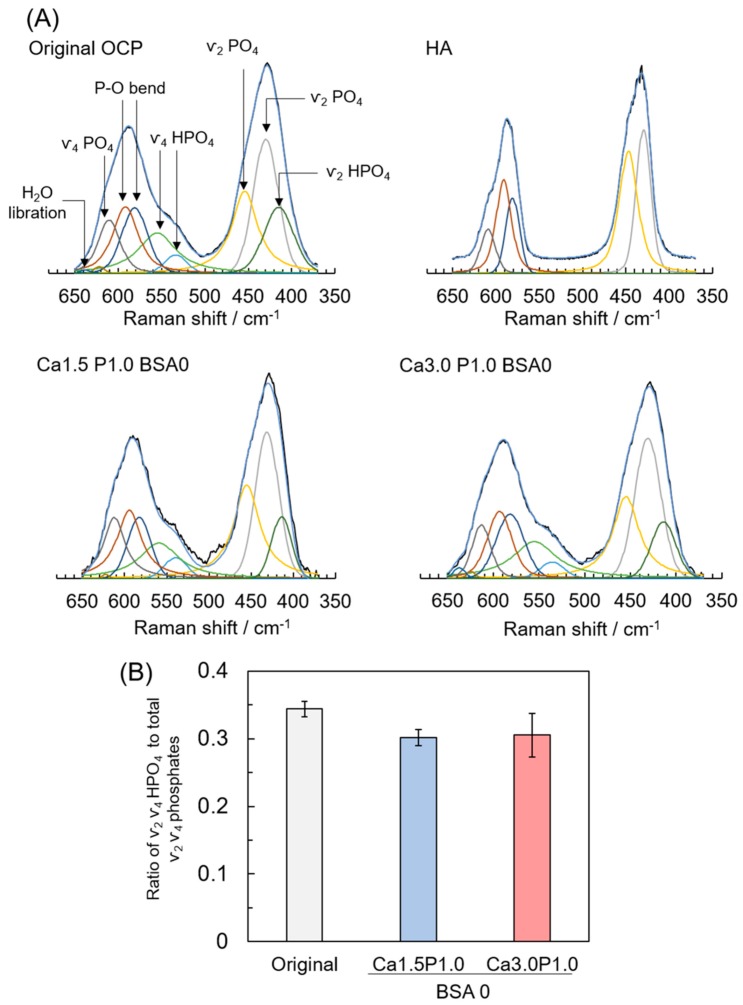
Raman spectra curve fitting of original OCP, hydroxyapatite (HA) and OCP after incubation with Ca1.5P1.0 or Ca3.0P1.0 solution without BSA (**A**). Integrated intensity ratio of ν_2_, ν_4_ HPO_4_ to the total phosphate vibration (ν_2_, ν_4_ HPO_4_ + ν_2_, ν_4_ PO_4_) estimated by a curve fitting of Raman spectra for OCP before and after incubation (**B**). Mean values were obtained from three independent experiments (n = 3).

To estimate the change in the HPO_4_^2−^ content of OCP during soaking in Ca1.5P1.0 and Ca3.0P1.0 solutions, a curve fitting of OCP Raman spectra was performed (Figure 6A). Peaks attributed to ν_2_ PO_4_ at 428 and 449 cm^−1^, ν_4_ PO_4_ at 608 cm^−1^ and the P–O bend at 582 and 593 cm^−1^ [44] were observed in the spectra of initial OCP and HA, which served as reference specimens. The peaks attributed to ν_2_ HPO_4_ at 417 cm^−1^ and ν_4_ HPO_4_ at 529 and 557 cm^−1^ [44] were detected in the spectra of the original OCP, but not in those for HA. The peaks attributed to ν_2_ and ν_4_ HPO_4_ vibration were also observed for OCP after soaking in Ca3.0P1.0 and Ca1.5P1.0 solutions without BSA. The integrated intensity ratio of ν_2_, ν_4_ HPO_4_ to the total phosphate vibration (i.e., ν_2_, ν_4_ HPO_4_ + ν_2_, ν_4_ PO_4_) between 370 and 650 cm^−1^ indicated that the HPO_4_^2−^ content in the calcium phosphate structure decreased from 0.34 to 0.30 or 0.31 after incubation in Ca1.5P1.0 or Ca3.0P1.0, respectively, without BSA for 1 h (Figure 6B), although the differences among the three groups were not statistically significant.

#### 3.3.3. TEM Observation

**Figure 7:**
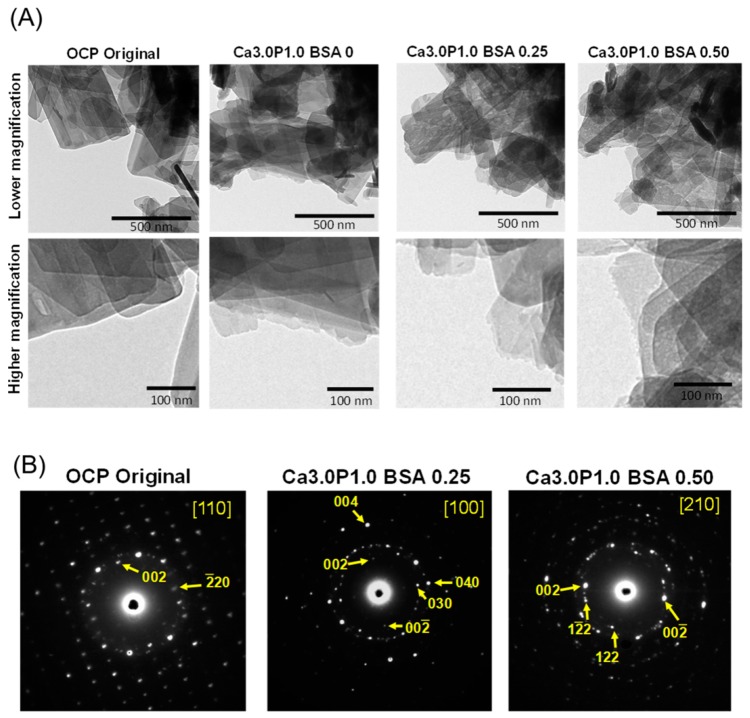
Bright field image of OCP before and after incubation with 0, 0.25 and 0.50 mg·mL^−1^ BSA in this Ca3.0P1.0 solution (**A**). Selected area of OCP electron diffraction before and after incubation in 0.25 or 0.50 mg·mL^−1^ BSA in the Ca3.0P1.0 solution (**B**).

In bright field images at lower magnification for original OCP, particles with a plate-like structure, which is a typical morphology for OCP crystals, were observed (Figure 7A). After incubation in Ca3.0P1.0 containing 0, 0.25 or 0.50 mg·mL^−1^ BSA, the plate-like structure was maintained, regardless of the BSA concentration.

Higher magnification revealed that the surface of the narrow side of the plate-like particle had small protrusions after incubation in Ca3.0P1.0 without BSA, in contrast to that seen for this side in the original OCP, which had a smooth surface. In addition, the narrow side of the plate was rougher for Ca3.0P1.0 with 0.25 or 0.50 mg·mL^−1^ BSA, compared to the sample incubated in the Ca3.0P1.0 solution without BSA.

To analyze the crystal structure of the plate particles observed in these bright field images, selected area electron diffraction (SAED) patterns were also obtained before and after soaking in the Ca3.0P1.0 solution containing BSA (Figure 7B). The SEAD pattern of the original OCP was assigned to the reflections along the [110] zone axis of OCP, which is a typical diffraction obtained from a single OCP crystal. In addition, the SEAD patterns of OCP after incubation in Ca3.0P1.0 with 0.25 and 0.50 mg·mL^−1^ BSA could be attributed to reflections along the [100] and [210] zone axis of OCP, which indicates that the plates maintained a crystal structure consistent with OCP after the incubation. These SAED patterns also showed that the plate-like particles oriented toward the c-axis and maintained the OCP crystal structure after the incubation.

### 3.4. Estimation of the Mass Ratio of Newly Formed OCP to OCP Granules after Incubation

**Figure 8:**
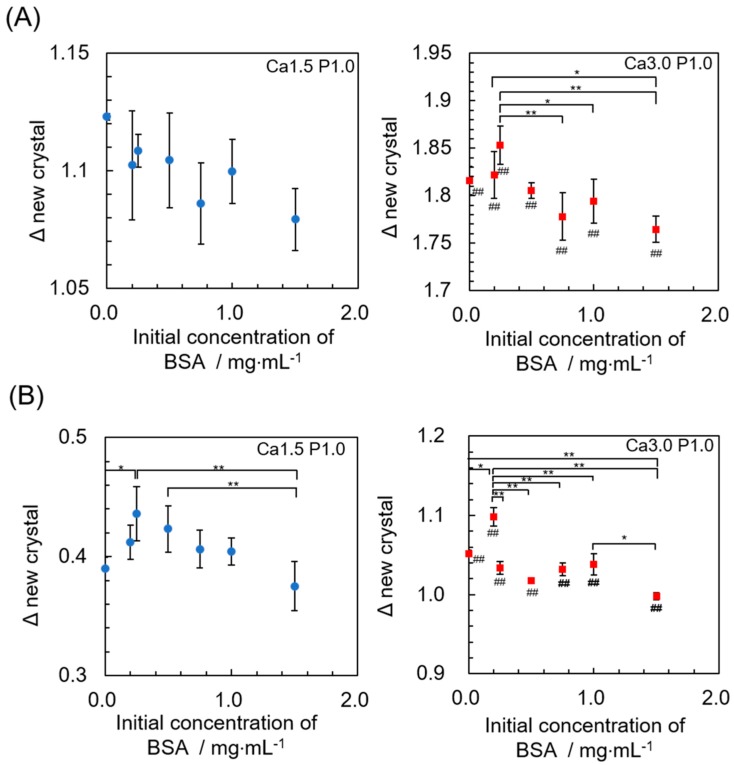
Change in the mass ratio of newly-formed calcium phosphate assuming that the OCP composition is the same as the original OCP determined from the decrease in the Ca^2+^ (**A**) and Pi ion (**B**) concentration after incubation in the Ca3.0P1.0 or Ca1.5P1.0 solution containing BSA. Mean values were obtained from three independent experiments (n = 3). *p < 0.05, **p < 0.01 significant difference among the BSA concentrations in each supersaturated solution. ^##^*p* < 0.01 significant difference between Ca1.5P1.0 and Ca3.0 P1.0 solution at each BSA concentration.

Changes in the amount of newly-formed calcium phosphate on the OCP crystals after incubation in both the Ca1.5P1.0 and the Ca3.0P1.0 solutions containing various amounts of BSA were determined based on decreases in Ca^2+^ or the Pi ion concentration, and estimations corresponding to newly-formed crystals assumed to have an OCP composition (Figure 8). The amount of newly-formed OCP was significantly higher in the Ca3.0P1.0 solution than the Ca1.5P1.0 solution, regardless of the BSA concentration. After incubation in either Ca1.5P1.0 or Ca3.0P1.0, the amount of newly-formed OCP tended to increase as the BSA concentration increased from 0 to 0.20 or 0.25 mg·mL^−1^. A significant increase in the amount of newly-formed OCP was observed, as estimated from the Pi ion concentration in both solutions after the incubations. Subsequently, the amount of newly-formed OCP significantly decreased with the increasing BSA concentration from 0.20 or 0.25 to 1.5 mg·mL^−1^, which was determined from the Ca^2+^ concentration in the Ca3.0P1.0 solution and the Pi ion in both solutions.

### 3.5. Mesurement of Ion Concentrations after the Incubation of OCP Granules with Pre-Adsorption of BSA in Supersaturated Solution

**Figure 9:**
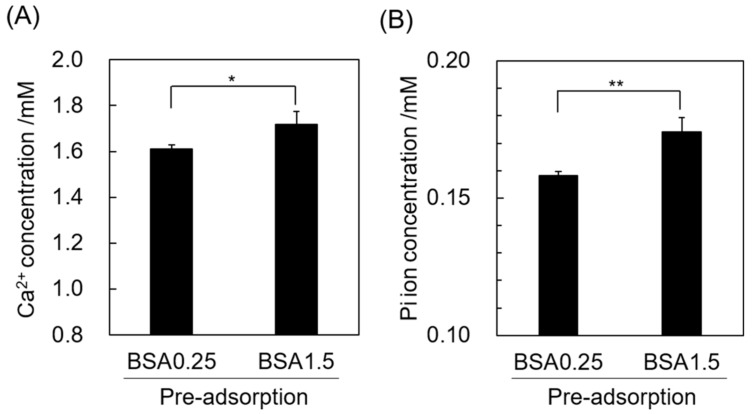
Ca^2+^ (**A**) and Pi ion (**B**) concentration in the Ca3.0P1.0 solution without BSA after incubation of OCP with pre-adsorbed BSA in the Ca3.0P1.0 solution containing 0.25 or 1.5 mg·mL^−1^ BSA. Mean values were obtained from three independent experiments (n = 3). *p < 0.05, **p < 0.01 significant difference.

Ca^2+^ and Pi ion concentrations in fresh Ca3.0P1.0 solution without BSA after the incubation with OCP granules with pre-adsorption of BSA were determined from adsorption experiments involving 0.25 and 1.5 mg·mL^−1^ BSA in the Ca3.0P1.0 solution (Figure 9). The concentration of both ions was significantly lower after the immersion of OCP granules in 0.25 mg·mL^−1^ BSA relative to 1.5 mg·mL^−1^ in fresh Ca3.0P1.0 solution.

## 4. Discussion

The present study examined the adsorption capacity of BSA on OCP in a 150 mM Tris-HCl buffer solution containing 1.5 or 3.0 mM Ca^2+^ and 1.0 mM Pi ion (Ca1.5P1.0 and Ca3.0P1.0, respectively) at pH 7.4, which represent supersaturated conditions with respect to HA and OCP. Results from the Bradford assay and Raman spectrometry revealed that increases in DS values around OCP granules enhanced the BSA adsorption onto the OCP surface (Figure 1 and Figure 5B). A previous study reported that the adsorption isotherm of BSA onto OCP crystals could be described using a Langmuir model in which the adsorption was measured under OCP saturation [25]. The Langmuir model equation is given in Equation (2):

Q = *KQ*_0_C/(1 + *K*C), (2)

where Q is the amount of adsorbate adsorbed on the adsorbent, C is the adsorbate equilibrium concentration, *K* is the equilibrium constant, and *Q*_0_ is the adsorbate saturation capacity. *K* and *Q*_0_ are 1520 mL·μmol^−1^ and 0.054 μmol·m^−2^, respectively, and were obtained from the previous adsorption isotherm [25]. Based on these parameters in the Langmuir equation, the previously reported adsorption isotherm was plotted in Figure 1B. The adsorption amount of the BSA rapidly increased at lower equilibrium concentrations of BSA. A local maximum value of Q for the supersaturated conditions relative to the saturated condition was detected, assuming that the behavior of the BSA adsorption isotherm at the higher DS could differ from that for the saturated condition. In fact, the adsorption isotherms in the supersaturated solutions did not correlate with the Langmuir model, because the correlation coefficient was < 0.9 when the data were plotted as 1/Q versus 1/C. Thus, these results suggested that the mechanism of BSA adsorption onto OCP differed under supersaturated and saturated conditions.

Decreases in Ca^2+^ and Pi ion concentration were observed after incubation of OCP in supersaturated solutions (Table 2), although the concentration of these ions remained relatively constant after incubation in the absence of OCP (Table 1). Under physiological conditions in vitro, the crystal structure of OCP can be converted into HA through a hydrolysis reaction in the presence of a fluoride ion [25]. Moreover, increases in DS with respect to HA that were supported by the presence of amorphous calcium phosphate promotes the conversion of OCP into HA in simulated body fluid (SBF) and osteogenic cell culture medium, even in the absence of fluoride ions [46,47]. However, the structural conversion does not always advance in SBF [48,49]. Yokoi et al. showed that after soaking in SBF for 14 days, new OCP formed on the OCP crystals prepared from CaCO_3_ and H_3_PO_4_ [48]. Ito et al. also reported that growth of plate-like OCP crystals occurred toward the long axis of the crystal in SBF that was replaced daily [49]. OCP crystals release Pi ions and incorporate Ca^2+^ in physiological solutions such as Tris buffer [14] and cell culture medium [50]. On the other hand, both Ca^2+^ and Pi ion concentrations decreased upon the precipitation of new OCP and/or crystal growth of OCP [48].

To confirm the structural change in OCP after incubation in Ca1.5P1.0 and Ca3.0P1.0, XRD, SAED and Raman spectroscopy analyses were performed. In XRD analysis for all specimens, the diffraction peak corresponding to (100) for OCP at 2θ = 4.7° remained, whereas the peak at 2θ = 11° corresponding to HA was not observed (Figure 3). SAED patterns obtained by TEM indicated that the crystal structure of plate-like OCP was maintained in Ca3.0P1.0 with BSA (Figure 7B). On the other hand, Raman spectroscopy detected characteristic HP(5)O_4_^2−^ that existed in the hydrated layer of the OCP structure (Figure 4), although the HPO_4_^2−^ ratio was slightly reduced (Figure 5) after the incubation. Suzuki et al. showed that a solid-solution exchange of HP(5)O_4_^2−^ occurs through diffusion in the hydrated layer and unstable HPO_4_^2−^ is irreversibly removed during hydrolysis at the surface of non-stoichiometric OCP during conversion to HA [51]. They also showed that the content of stable HP(6)O_4_^2−^, exchangeable HP(5)O_4_^2−^ and unstable HPO_4_^2−^ are 50–60%, 25–30% and 15–20%, respectively, with respect to total phosphate in OCP [51]. Semiquantitative analysis using curve fitting estimated that the amount of HPO_4_^2−^ in the total phosphate declined by about 11% after the incubation (Figure 6B). The percentage of HPO_4_^2−^ in OCP synthesized by wet synthesis, as was carried out in this study, was previously determined by chemical analysis to be 36%–40% [50,51]. Our Raman spectroscopy suggested that the unstable HPO_4_^2−^ tended to be removed and HP(5)O_4_^2−^ could be partially exchanged after incubation in the Ca3.0P1.0 and Ca1.5P1.0 solutions. Taken together, the diffusion of HPO_4_^2−^ in the hydrated layer of OCP would be insufficient to substantially convert into an HA structure in Ca1.5P1.0 and Ca3.0P1.0, both with and without BSA.

TEM bright field images showed that the plate-like OCP crystals had small terminal protrusions after soaking in Ca3.0P1.0 with and without BSA (Figure 7A), although in the presence of BSA the protrusions were finer. The SAED patterns indicated that such protrusions could form on the c-plane of OCP plate crystals (Figure 7B). In fact, a similar shape on the edges of plate-like OCP crystals was previously observed when new OCP crystals with flake-like shapes formed on the plate [48]; these plate-like crystals grew toward the long-axis [49] after immersion in SBF. Thus, the change in the shape of the terminal protrusions observed here was likely associated with new OCP formation and the crystal growth of OCP in the supersaturated solution. On the other hand, the calculated DS values (Table 2) indicated that the OCP phase was thermodynamically metastable after incubation in the solutions, regardless of the initial DS value and the BSA concentration. This result suggests that the newly-formed crystals in such solutions could also be metastable OCP. Based on the TEM results and DS calculations, higher DS could provide a driving force for the nucleation and crystal growth of new OCP on existing OCP crystals in both the presence and absence of BSA. Taken together, it is reasonable to assume that the growth of new OCP crystals may be preferentially induced by supersaturated conditions in the presence of BSA rather than by structural conversion into HA, which enhanced the decrease in the Ca^2+^ and Pi ion concentration.

The mass ratio of newly formed calcium phosphate estimated as OCP to the original OCP increased with increases from the initial DS values in the BSA solution (Figure 8), which were calculated as decreases in the amount of Ca^2+^ and Pi ions in the solutions (Table 2). This tendency was similar to the increase in the amount of BSA adsorbed with increasing DS values. Crystal growth sites for HA are suggested to act as sites for the adsorption of acidic proteins [52]. In addition, calcium phosphates are reported to incorporate proteins during precipitation [53,54,55]. In terms of OCP, the incorporation of enamel proteins [56] and natural polymers, such as gelatin, was shown to occur thorough OCP precipitation and crystal growth [33,57]. The affinity of an adsorbent for an adsorbate is generally accepted to influence the adsorption behavior of molecules, particularly at lower adsorbate concentrations, which corresponds to the equilibrium constant *K* in the Langmuir model. Our previous studies demonstrate that the crystal phase and composition of calcium phosphate (OCP, HA and fluoridated apatite) influence the affinity for proteins such as BSA and human serum albumin [25,58]. The results of the present study demonstrated that the adsorption of BSA onto OCP was promoted to a greater degree by supersaturated conditions relative to that for saturated solutions as was previously seen for lower BSA concentrations (Figure 1B). Although the amount of adsorbed BSA was higher for Ca3.0P1.0 than Ca1.5P1.0 after incubation, the structural convection was not confirmed and the HPO_4_^2−^ ratio estimated by Raman curve fitting suggested that OCP incubated in Ca1.5P1.0 and Ca3.0P1.0 had a similar composition (Figure 6). According to these results, a higher initial DS could increase the number of adsorption sites and the coprecipitation of BSA with OCP could promote the formation and growth of new OCP crystals, resulting in enhanced adsorption at lower BSA concentrations.

In contrast, higher concentrations of BSA, 0.75–1.5 mg·mL^−1^, reduced not only the amount of BSA adsorbed, but also formation of new OCP induced by increased DS. For HA, other research groups reported that free proteins in the solution negatively affected the HA crystal growth [59]. To clarify how the higher BSA concentration affects OCP formation in supersaturated solutions, here we estimated the free Ca^2+^ concentration (Figure 2). Free Ca^2+^ tended to decrease for both Ca1.5P1.0 and Ca3.0P1.0 as the BSA concentration increased. Serum albumin has been reported to bind Ca^2+^ under physiological conditions [60,61]. Pedersen et al. reported that the association constant was 90–100 L·mol^−1^ for albumin bound to Ca^2+^ at pH 7.4 under ionic strengths ranging from 0.15 to 0.16 M at 37 °C [60]. Thus, at higher BSA concentrations, an increased frequency of BSA and Ca^2+^ interactions could reduce the supply of free Ca^2+^ available for OCP crystal growth. On the other hand, Combes et al. reported that BSA can occupy active growth sites to inhibit the diffusion of ions that could contribute to OCP crystal growth on collagen-coating substrates in the presence of higher BSA concentrations [62,63]. In this study, decreases in the Ca^2+^ and Pi ion concentrations in fresh Ca3.0P1.0 solution without BSA were smaller for OCP that was pre-adsorbed with BSA at 1.5 mg·mL^−1^ relative to 0.25 mg·mL^−1^ BSA (Figure 9), indicating that the pre-adsorbed BSA could decrease Ca^2+^ and Pi ion uptake for OCP crystal growth. This result also suggested that larger amounts of BSA could mask OCP surfaces in the higher concentration of BSA compared to the lower concentrations during the initial incubation, in turn delaying the crystal growth of adsorbent OCP in supersaturated solutions. The higher concentration of BSA could thus suppress crystal growth through one or both of these two mechanisms, resulting in the decrease in the adsorption amount due to the inhabitation of the new adsorption site formation and the coprecipitation.

Lower BSA concentrations (0.20–0.25 mg·mL^−1^) could increase the rate of new OCP formation relative to that in the presence of supersaturated solutions lacking BSA. Similar phenomena were observed in previous studies wherein the growth rate of OCP on a type I collagen coating was faster in supersaturated solutions containing lower BSA concentrations than in the absence of BSA [62,63]. These studies proposed that BSA promotes OCP nucleation and subsequent crystal growth on the collagen under such conditions [62,63]. Ijma et al. also demonstrated that human serum albumin adsorbed on a synthetic polymer could induce a heterogeneous nucleation of calcium phosphates such as apatite in a solution having ion concentrations 1.5-fold higher than that of SBF [64]. On the other hand, another group reported that OCP crystal growth on OCP seed crystals involved polynucleation [65]. Based on these previous reports, our results suggested that the number of crystal growth sites is increased by nucleation induced by adsorbed BSA to promote the growth of new OCP crystals on existing OCP at the lower BSA concentration in Ca1.5P1.0 and Ca3.0P1.0 solutions.

Although formation of new OCP was enhanced at 0.20–0.25 mg·mL^−1^ BSA (Figure 8), the adsorption amount was maximized at 0.50 mg·mL^−1^ BSA (Figure 1A). In the solutions containing 0.20–0.25 mg·mL^−1^ BSA, the adsorption isotherm showed that BSA was nearly completely adsorbed onto the OCP, which suggests that most BSA was incorporated into the newly-formed OCP. Thus, the additional adsorption afforded at 0.50 mg·mL^−1^ BSA was less effective at inhibiting OCP formation than higher concentrations of BSA.

Finally, the results of the present study revealed that higher DS with respect to HA and OCP could promote BSA adsorption on OCP through the nucleation and crystal growth of newly formed OCP after a brief incubation in supersaturated solutions. Previous studies demonstrated that osteoblastic mineralization was induced on OCP crystals where fine crystals are being formed during the transformation into HA and serum glycoproteins accumulate on mouse calvaria [7,15,20]. If the findings in the present study indeed simulate the initial stage of the reprecipitation reaction after OCP dissolution, then the protein-OCP coprecipitation on OCP crystals may mimic the accumulation of proteins and subsequent bone mineralization that occurs in vivo. However, the adsorption behavior of only one type of protein was investigated in the present study. In future work, the relationship between the selective adsorption of serum proteins on OCP and crystal growth during the structural transformation should be examined in additional detail to establish the linkage between in vitro and in vivo conditions.

## 5. Conclusions

The effects of the degree of supersaturation (DS) with respect to OCP and HA on BSA adsorption onto OCP were examined. Increases in the DS were associated with an increased adsorption capacity of BSA and newly formed calcium phosphate, which could possibly be metastable OCP, onto OCP. In addition, the formation of new calcium phosphate inhibited by Ca^2+^ binding to BSA in the solution and masking of crystal growth sites on the OCP was related to a decreased adsorption capacity at higher BSA concentrations. Results from this study suggest that the formation of new calcium phosphate crystals, which is regulated by both DS and the protein concentration, is a key factor for the accumulation of serum protein on the OCP surface under physiological conditions.

## Figures and Tables

**Table 1:** Concentration of Ca^2+^ and Pi ions in Ca1.5P1.0 and Ca3.0P1.0 solution before and after 1 h of incubation without OCP.

Sample	Ca^2+^/mM ^a^	Pi ion/mM ^a^	pH
Original Ca1.5P1.0	1.49	1.00	7.4
Ca1.5P1.0 BSA0	1.52	1.00	7.4
Ca1.5P1.0 BSA0.20	1.51	0.996	7.4
Ca1.5P1.0 BSA 0.25	1.51	0.955	7.4
Ca1.5P1.0 BSA0.50	1.48	0.975	7.4
Ca1.5P1.0 BSA0.75	1.47	0.975	7.4
Ca1.5P1.0 BSA1.0	1.50	0.991	7.4
Ca1.5P1.0 BSA1.5	1.47	0.981	7.4
Original Ca3.0P1.0	3.07	0.987	7.4
Ca3.0P1.0 BSA0	3.05	0.995	7.4
Ca3.0P1.0 BSA0.20	3.00	1.00	7.4
Ca3.0P1.0 BSA0.25	3.02	0.965	7.4
Ca3.0P1.0 BSA0.50	3.02	0.960	7.4
Ca3.0P1.0 BSA0.75	3.07	0.975	7.4
Ca3.0P1.0 BSA1.0	3.02	0.97	7.4
Ca3.0P1.0 BSA1.5	3.09	0.95	7.4

^a^ Average values obtained from 3 independent experiments.

**Table 2:** Composition and degree of supersaturation (DS) of Ca1.5P1.0 and Ca3.0 P1.0 solution before and after incubation with OCP for 1 h.

Sample	Ca^2+^/ mM ^a^	Pi ion/ mM ^a^	pH	DS at 37 °C (150 mM Tris-HCl)		
				HA	OCP	DCPD
Original Ca1.5P1.0	1.49	1.00	7.4	1.56 × 10^11^	581	4.70 × 10^−1^
Ca1.5P1.0 BSA0	0.503	0.737	7.4	3.16 × 10^8^	3.45	1.22 × 10^−1^
Ca1.5P1.0 BSA0.20	0.512	0.722	7.4	3.25 × 10^8^	3.48	1.22 × 10^−1^
Ca1.5P1.0 BSA0.25	0.500	0.705	7.4	2.73 × 10^8^	2.98	1.16 × 10^−1^
Ca1.5P1.0 BSA0.50	0.491	0.714	7.4	2.59 × 10^8^	2.88	1.16 × 10^−1^
Ca1.5P1.0 BSA0.75	0.511	0.726	7.4	3.27 × 10^8^	3.51	1.22 × 10^−1^
Ca1.5P1.0 BSA1.0	0.500	0.727	7.4	2.95 × 10^8^	3.24	1.20 × 10^−1^
Ca1.5P1.0 BSA1.5	0.518	0.747	7.4	3.80 × 10^8^	4.02	1.27 × 10^−1^
Original Ca3.0P1.0	3.07	0.987	7.4	4.57 × 10^12^	8.30 × 10^3^	8.99 × 10^−1^
Ca3.0P1.0 BSA0	1.39	0.285	7.4	2.86 × 10^9^	11.1	1.28 × 10^−1^
Ca3.0P1.0 BSA 0.20	1.36	0.257	7.4	1.89 × 10^9^	7.54	1.13 × 10^−1^
Ca3.0P1.0 BSA0.25	1.35	0.263	7.4	1.93 × 10^9^	7.76	1.15 × 10^−1^
Ca3.0P1.0 BSA0.50	1.38	0.259	7.4	2.03 × 10^9^	8.00	1.15 × 10^−1^
Ca3.0P1.0 BSA0.75	1.40	0.273	7.4	2.60 × 10^9^	10.1	1.23 × 10^−1^
Ca3.0P1.0 BSA1.0	1.39	0.267	7.4	2.32 × 10^9^	9.08	1.20 × 10^−1^
Ca3.0P1.0 BSA1.5	1.41	0.270	7.4	2.64 × 10^9^	10.1	1.23 × 10^−1^

^a^ Average values obtained from 3 independent experiments.

## References

[1] Brown W.E. (1966). Crystal growth of bone mineral. Clin. Orthop. Relat. Res..

[2] Brown W.E., Smith J.P., Frazier A.W., Lehr J.R. (1962). Crystallographic and chemical relations between octacalcium phosphate and hydroxyapatite. Nature.

[3] Brown W.E., Mathew M., Tung M.S. (1981). Crystal chemistry of octacalcium phosphate. Prog. Cryst. Growth Charact..

[4] Meyer J.L., Eanes E.D. (1978). Thermodynamic analysis of secondary transition in spontaneous precipitation of calcium phosphate. Calcif. Tissue Res..

[5] Chow L.C. (2009). Next generation calcium phosphate-based biomaterials. Dent. Mater. J..

[6] Lu X., Leng Y. (2005). Theoretical analysis of calcium phosphate precipitation in simulated body fluid. Biomaterials.

[7] Suzuki O., Nakamura M., Miyasaka Y., Kagayama M., Sakurai M. (1991). Bone formation on synthetic precursors of hydroxyapatite. Tohoku J. Exp. Med..

[8] Kikawa T., Kashimoto O., Imaizumi H., Kokubun S., Suzuki O. (2009). Intramembranous bone tissue response to biodegradable octacalcium phosphate implant. Acta Biomater..

[9] Imaizumi H., Sakurai M., Kashimoto O., Kikawa T., Suzuki O. (2006). Comparative study on osteoconductivity by synthetic octacalcium phosphate and sintered hydroxyapatite in rabbit bone marrow. Calcif. Tissue Int..

[10] Suzuki O., Kamakura S., Katagiri T., Nakamura M., Zhao B.H., Honda Y., Kamijo R. (2006). Bone formation enhanced by implanted octacalcium phosphate involving conversion into Ca-deficient hydroxyapatite. Biomaterials.

[11] Anada T., Araseki A., Matsukawa S., Yamasaki T., Kamakura S., Suzuki O. (2008). Effect of Octacalcium Phosphate Ionic Dissolution Products on Osteoblastic Cell Differentiation.

[12] Takami M., Mochizuki A., Yamada A., Tachi K., Zhao B., Miyamoto Y., Anada T., Honda Y., Inoue T., Nakamura M. (2009). Osteoclast differentiation induced by synthetic octacalcium phosphate through receptor activator of NF-κ B ligand expression in osteoblasts. Tissue Eng. Part. A.

[13] Lotsari A., Rajasekharan A.K., Halvarsson M., Andersson M. (2018). Transformation of amorphous calcium phosphate to bone-like apatite. Nat. Commun..

[14] Suzuki O., Kamakura S., Katagiri T. (2006). Surface chemistry and biological responses to synthetic octacalcium phosphate. J. Biomed. Mater. Res. B Appl. Biomater..

[15] Suzuki O., Nakamura M., Miyasaka Y., Kagayama M., Sakurai M. (1993). Maclura pomifera agglutinin-binding glycoconjugates on converted apatite from synthetic octacalcium phosphate implanted into subperiosteal region of mouse calvaria. Bone Miner..

[16] Suzuki Y., Kamakura S., Honda Y., Anada T., Hatori K., Sasaki K., Suzuki O. (2009). Appositional bone formation by OCP-collagen composite. J. Dent. Res..

[17] Aoba T., Shimazu Y., Taya Y., Soeno Y., Sato K., Miake Y. (2003). Fluoride and apatite formation in vivo and in vitro. J. Electron. Microsc..

[18] Kerschnitzki M., Akiva A., Ben Shoham A., Asscher Y., Wagermaier W., Fratzl P., Addadi L., Weiner S. (2016). Bone mineralization pathways during the rapid growth of embryonic chicken long bones. J. Struct. Biol..

[19] Kaneko H., Kamiie J., Kawakami H., Anada T., Honda Y., Shiraishi N., Kamakura S., Terasaki T., Shimauchi H., Suzuki O. (2011). Proteome analysis of rat serum proteins adsorbed onto synthetic octacalcium phosphate crystals. Anal. Biochem..

[20] Suzuki O., Imaizumi H., Kamakura S., Katagiri T. (2008). Bone regeneration by synthetic octacalcium phosphate and its role in biological mineralization. Curr. Med. Chem..

[21] Eidelman N., Chow L.C., Brown W.E. (1987). Calcium phosphate saturation levels in ultrafiltered serum. Calcif. Tissue Int..

[22] Shibuya I., Yoshimura K., Miyamoto Y., Yamada A., Takami M., Suzawa T., Suzuki D., Ikumi N., Hiura F., Anada T. (2013). Octacalcium phosphate suppresses chondrogenic differentiation of ATDC5 cells. Cell Tissue Res..

[23] Hirayama B., Anada T., Shiwaku Y., Miyatake N., Tsuchiya K., Nakamura M., Takahashi T., Suzuki O. (2016). Immune cell response and subsequent bone formation induced by implantation of octacalcium phosphate in a rat tibia defect. RSC Adv..

[24] Ea H.K., Uzan B., Rey C., Liote F. (2005). Octacalcium phosphate crystals directly stimulate expression of inducible nitric oxide synthase through p38 and JNK mitogen-activated protein kinases in articular chondrocytes. Arthritis Res. Ther..

[25] Suzuki O., Yagishita H., Yamazaki M., Aoba T. (1995). Adsorption of bovine serum albumin onto octacalcium phosphate and its hydrolyzates. Cells Mater..

[26] Aoba T., Moreno E.C., Shimoda S. (1992). Competitive adsorption of magnesium and calcium-ions onto synthetic and biological apatites. Calcif. Tissue Int..

[27] Moreno E.C., Kresak M., Hay D.I. (1984). Adsorption of molecules of biological interest onto hydroxyapatite. Calcif. Tissue Int..

[28] Lee H.S., Myers C., Zaide L., Nalam P.C., Caporizzo M.A., Daep C.A., Eckmann D.M., Masters J.G., Composto R.J. (2017). Competitive adsorption of polyelectrolytes onto and into pellicle-coated hydroxyapatite investigated by QCM-D and force spectroscopy. ACS Appl. Mater. Interfaces.

[29] Moreno E.C., Varughese K., Hay D.I. (1979). Effect of human salivary proteins on the precipitation kinetics of calcium phosphate. Calcif. Tissue Int..

[30] Lamkin M.S., Arancillo A.A., Oppenheim F.G. (1996). Temporal and compositional characteristics of salivary protein adsorption to hydroxyapatite. J. Dent. Res..

[31] Komlev V.S., Barinov S.M., Bozo I.I., Deev R.V., Eremin I.I., Fedotov A.Y., Gurin A.N., Khromova N.V., Kopnin P.B., Kuvshinova E.A. (2014). Bioceramics composed of octacalcium phosphate demonstrate enhanced biological behavior. ACS Appl. Mater. Interfaces.

[32] Kawai T., Echigo S., Matsui K., Tanuma Y., Takahashi T., Suzuki O., Kamakura S. (2014). First clinical application of octacalcium phosphate collagen composite in human bone defect. Tissue Eng. Part. A.

[33] Handa T., Anada T., Honda Y., Yamazaki H., Kobayashi K., Kanda N., Kamakura S., Echigo S., Suzuki O. (2012). The effect of an octacalcium phosphate co-precipitated gelatin composite on the repair of critical-sized rat calvarial defects. Acta Biomater..

[34] Chiba S., Anada T., Suzuki K., Saito K., Shiwaku Y., Miyatake N., Baba K., Imaizumi H., Hosaka M., Itoi E. (2016). Effect of resorption rate and osteoconductivity of biodegradable calcium phosphate materials on the acquisition of natural bone strength in the repaired bone. J. Biomed. Mater. Res. Part. A.

[35] Medvecky L., Sopcak T. (2012). Preparation and properties of octacalcium phosphate-polyhydroxybutyrate thin film composites. Mater. Lett..

[36] Heydari Z., Mohebbi-Kalhori D., Afarani M.S. (2017). Engineered electrospun polycaprolactone (PCL)/octacalcium phosphate (OCP) scaffold for bone tissue engineering. Mater. Sci. Eng. C Mater. Biol. Appl..

[37] Birgani Z.T., Malhotra A., van Blitterswijk C.A., Habibovic P. (2016). Human mesenchymal stromal cells response to biomimetic octacalcium phosphate containing strontium. J. Biomed. Mater. Res. Part. A.

[38] Hiromoto S., Inoue M., Taguchi T., Yamane M., Ohtsu N. (2015). In vitro and in vivo biocompatibility and corrosion behaviour of a bioabsorbable magnesium alloy coated with octacalcium phosphate and hydroxyapatite. Acta Biomater..

[39] Moreno E.C., Margolis H.C. (1988). Composition of human plaque fluid. J. Dent. Res..

[40] Aoba T., Moreno E.C. (1987). The enamel fluid in the early secretory stage of porcine amelogenesis: Chemical composition and saturation with respect to enamel mineral. Calcif. Tissue Int..

[41] Moreno E.C., Kresak M., Zahradni R.T. (1974). Fluoridated hydroxyapatite solubility and caries formation. Nature.

[42] Tung M.S., Eidelman N., Sieck B., Brown W.E. (1988). Octacalcium phosphate solubility product from 4 to 37 °C. J. Res. Natl. Bur. Stand..

[43] Moreno E.C., Brown W.E., Osborn G. (1960). Solubility of dicalcium phosphate dihydrate in aqueous systems I. Soil Sci. Soc. Am. J..

[44] Fowler B.O., Markovic M., Brown W.E. (1993). Octacalcium phosphate. 3. infrared and raman vibrational-spectra. Chem. Mater..

[45] Chen M.C., Lord R.C. (1976). laser-excited raman-spectroscopy of biomolecules. VIII. conformational study of bovine serum-albumin. J. Am. Chem. Soc..

[46] Kobayashi K., Anada T., Handa T., Kanda N., Yoshinari M., Takahashi T., Suzuki O. (2014). Osteoconductive property of a mechanical mixture of octacalcium phosphate and amorphous calcium phosphate. ACS Appl. Mater. Interfaces.

[47] Sato T., Anada T., Hamai R., Shiwaku Y., Tsuchiya K., Sakai S., Baba K., Sasaki K., Suzuki O. (2019). Culture of hybrid spheroids composed of calcium phosphate materials and mesenchymal stem cells on an oxygen-permeable culture device to predict in vivo bone forming capability. Acta Biomater..

[48] Yokoi T., Kim I.Y., Ohtsuki C. (2012). Mineralization of calcium phosphate on octacalcium phosphate in a solution mimicking in vivo conditions. Phosphorus Res. Bull..

[49] Ito N., Kamitakahara M., Yoshimura M., Ioku K. (2014). Importance of nucleation in transformation of octacalcium phosphate to hydroxyapatite. Mater. Sci. Eng. C Mater. Biol. Appl..

[50] Sai Y., Shiwaku Y., Anada T., Tsuchiya K., Takahashi T., Suzuki O. (2018). Capacity of octacalcium phosphate to promote osteoblastic differentiation toward osteocytes in vitro. Acta Biomater..

[51] Suzuki O., Yagishita H., Amano T., Aoba T. (1995). Reversible structural changes of octacalcium phosphate and labile acid phosphate. J. Dent. Res..

[52] Aoba T., Moreno E.C. (1985). Adsorption of phosphoserine onto hydroxyapatite and its inhibitory activity on crystal-growth. J. Colloid Interface Sci..

[53] Wen H.B., de Wijn J.R., van Blitterswijk C.A., de Groot K. (1999). Incorporation of bovine serum albumin in calcium phosphate coating on titanium. J. Biomed. Mater. Res..

[54] Oyane A., Wang X.P., Sogo Y., Ito A., Tsurushima H. (2012). Calcium phosphate composite layers for surface-mediated gene transfer. Acta Biomater..

[55] Uchida M., Oyane A., Kim H.M., Kokubo T., Ito A. (2004). Biomimetic coating of laminin-apatite composite on titanium metal and its excellent cell-adhesive properties. Adv. Mater..

[56] Moradian-Oldak J. (2001). Amelogenins: assembly, processing and control of crystal morphology. Matrix Biol..

[57] Ishiko-Uzuka R., Anada T., Kobayashi K., Kawai T., Tanuma Y., Sasaki K., Suzuki O. (2017). Oriented bone regenerative capacity of octacalcium phosphate/gelatin composites obtained through two-step crystal preparation method. J. Biomed. Mater. Res. Part. B Appl. Biomater..

[58] Tsutsui S., Anada T., Shiwaku Y., Tsuchiya K., Yamazaki H., Suzuki O. (2018). Surface reactivity of octacalcium phosphate-derived fluoride-containing apatite in the presence of polyols and fluoride. J. Biomed. Mater. Res. Part. B Appl. Biomater..

[59] Lussi A., Crenshaw M.A., Linde A. (1988). Induction and inhibition of hydroxyapatite formation by rat dentin phosphoprotein invitro. Arch. Oral Biol..

[60] Pedersen K.O. (1971). binding of calcium to serum albumin. I. Stoichiometry and intrinsic association constant at physiological ph, ionic strength, and temperature. Scand. J. Clin. Lab. Investig..

[61] Carr C.W. (1953). Studies on the binding of small ions in protein solutions with the use of membrane electrodes. II. The binding of calcium ions in solutions of bovine serum albumin. Arch. Biochem. Biophys..

[62] Combes C., Rey C., Freche M. (1999). In vitro crystallization of octacalcium phosphate on type I collagen: influence of serum albumin. J. Mater. Sci. Mater. Med..

[63] Combes C., Rey C. (2002). Adsorption of proteins and calcium phosphate materials bioactivity. Biomaterials.

[64] Iijima K., Sakai A., Komori A., Sakamoto Y., Matsuno H., Serizawa T., Hashizume M. (2015). Control of biomimetic hydroxyapatite deposition on polymer substrates using different protein adsorption abilities. Colloids Surf. B-Biointerfaces.

[65] Heughebaert J.C., Nancollas G.H. (1984). Kinetics of crystallization of octacalcium phosphate. J. Phys. Chem..

